# Decoding diversity: Are there patterns in the organization of grooming movements in domestic cats?

**DOI:** 10.1002/jeab.70146

**Published:** 2026-10-01

**Authors:** Anna Hock, Manuela Schmidt

**Affiliations:** ^1^ Institute of Zoology and Evolutionary Research, Friedrich Schiller University Jena Germany

**Keywords:** analysis of intraspecific variation, grooming performance, postural flexibility, sequencing pattern

## Abstract

Grooming is crucial for mammalian health and hygiene, serving diverse functions such as parasite defense, removing dirt, and thermoregulation. Grooming is not purely reactive but integrated into daily routines and regulated endogenously. In rodents, it has been shown to follow a sequential structure with predictable patterns, often interpreted as reflecting pattern‐generating neurological programs. In this study, we examined the temporal and spatial structure of self‐grooming sequences in cats to test whether grooming in this species follows similarly predictable patterns. Grooming performance in 17 cats was recorded, and 40 sequences were analyzed to discern the allocation of grooming time to different body areas. Pattern analyses reveal the temporal order in which body areas were cleaned. Oral grooming occupies the largest portion of grooming time, whereas face washing and scratching were less frequent. High individual variation was found in the distribution of grooming time across body areas and in the spatiotemporal order of sequences, challenging the idea of strict patterns and grooming rules in cats. Instead, our analyses indicate that grooming is structured by probabilistic transitions between spatially adjacent body regions. We conclude that grooming sequences in cats are primarily determined by the spatial proximity of grooming targets and movement efficiency.

Grooming behavior and performance have been extensively studied, especially in mammals and birds (e.g., Alt et al., 2020; Berridge, 1990; Borchelt et al., 1976; Giles & Russell, 1974; Goodman et al., 2020; Hart, 1990; Lefebvre & Joly, 1982; McManus, 1970; Mooring et al., 2000; Moreno‐Rueda, 2017; Richmond & Sachs, 1980; Spruijt et al., 1992) but have also been observed in many invertebrate species (e.g., Barrientos, 2020; Basibuyuk & Quicke, 1999; Chirivi Joya et al., 2021; Coppard et al., 2012; Engel, 2012; Kuo et al., 2022; Lefebvre, 1981; Valentine, 1973; Wortham & Pascual, 2017). Grooming can generally be classified into two categories: self‐grooming or *autogrooming* in which individuals clean their own body using the mouth and/or limbs and *allogrooming* in which conspecifics groom each other. The relative importance of these options varies across species and ecological contexts, and they differ in their functional roles, organization, and associated motor demands.

The functions attributed to grooming are diverse and vary depending on the species. For example, its importance for parasite defense depends on the species‐specific vulnerability to infestation, which is influenced by the animal's size: Smaller animals with their larger surface‐to‐volume ratio can tolerate fewer parasites per unit of body surface (Amador & Hu, 2015). The typical habitat for the species also influences the risk of parasitic infestation (Hart, 1990; Hart et al., 1992; Mooring et al., 2000). The cleaning function of grooming is especially important for small animals, such as insects, where removing dirt is essential for maintaining mobility (e.g., Amador & Hu, 2015; Wortham & LaVelle, 2016). However, keeping the body surface clean is also relevant for larger animals, such as mammals and birds, as it helps maintain vital senses such as touch and hearing and prevents infections (Amador & Hu, 2015; Hart, 1990). Oral grooming, as found in mammals, also plays a role in thermoregulation, due to the cooling effect resulting from the evaporating saliva (Noel & Hu, 2018; Roberts, 1988; Spruijt et al., 1992). The importance of grooming is further reflected in the associated costs, which manifest in the form of high time and energy expenditure, reduced vigilance against predators, and water loss during oral grooming (Giorgi et al., 2001; Hart, 1990; Hawlena et al., 2007). Despite these high costs, the behavior is widespread, suggesting a selective advantage associated with efficient and effective grooming. In addition to the delayed functional benefits, grooming may be maintained by its immediate sensory consequences, allowing it to function as a natural reinforcer (Kalueff et al., 2016; Spruijt et al., 1992).

Domestic cats dedicate up to 24% of their waking time to self‐grooming (Eckstein & Hart, 2000a; Swenson & Randall, 1977; Watanabe et al., 2005), comparable to levels reported in rats (25%–40%; Bolles, 1960). In most mammals, self‐grooming consists of three components: oral grooming using teeth and tongue; face washing using wipes of the dry or wet fore paws; and scratching, mostly with the hind paws (Fentress & Stilwell, 1973; McManus, 1970; Richmond & Sachs, 1980). According to previous studies, most of the grooming activity in cats is attributed to oral grooming, whereas face washing using the fore paws and scratching consume less time (Eckstein & Hart, 2000a; Watanabe et al., 2005).

The prevalence of ectoparasites highlights the importance of grooming for cats (Eckstein & Hart, 2000b; Thomas et al., 2016). Estimates for flea infestation vary from 15% to over 70% depending on the region and the animals' living condition, and ticks infest 20% to 30% of free‐roaming cats (Beck et al., 2006; Bond et al., 2007; Beugnet et al., 2014; Thomas et al., 2016). These parasites can transmit a variety of diseases and cause infections (Lappin, 2018; Sotiraki et al., 2001), highlighting the importance of oral and scratch grooming to prevent and eliminate infestation. Although the level of grooming activity in mammals increases with parasitic infestation, this increase includes mostly stimulus‐driven and localized grooming (Giorgi et al., 2001; Hawlena et al., 2007). Grooming sequences covering multiple body areas are likely innate, endogenously regulated, and to a certain degree stereotyped (Berridge, 1990; Berridge & Fentress, 1987). In rodents, a grooming sequence typically consists of consecutive episodes dedicated to different body regions (Berridge, 1990; Berridge et al., 1987; Fentress & Stilwell, 1973; Richmond & Sachs, 1980). These patterns reveal a hierarchical structure, termed syntactic chain, where individual elements of grooming can be structured into higher level units according to rules rather than being randomly arranged (Berridge et al., 1987). An endogenous regulation via a neuronal central pattern generator has been frequently suggested based on the observation of these predictable movement patterns (Berridge & Fentress, 1987; Eckstein & Hart, 2000a; Fentress & Stilwell, 1973; Giles & Russell, 1974; Hart, 1990; Lefebvre, 1981, 1982; Lefebvre & Joly, 1982; Swenson & Randall, 1977; Richmond & Sachs, 1980). A cephalocaudal progression of grooming sequences as part of these rules has been described for various rodents (Berridge, 1990; Fentress & Stilwell, 1973), opossums, cats (Eckstein & Hart, 2000a; McManus, 1970; Swenson & Randall, 1977), birds, (Lefebvre, 1982; Lefebvre and Joly, 1982), and even insects (Lefebvre, 1981). While endogenous regulation certainly plays an essential role in grooming, a problematic assumption arises when this regulation is automatically equated with the presence of strict, deterministic rules. Much of the classical work has taken a hypothesis‐driven confirmatory approach, seeking to prove the existence of preestablished rules or rigid patterns of grooming organization (e.g., Berridge, 1990; Berridge, et al. 1987; Eckstein & Hart, 2000a; Lefebvre & Joly, 1982).

The present study takes an exploratory approach focused on quantifying intraspecific and intraindividual variation in grooming organization to characterize species‐specific performance. Based on a sample of 17 individual cats of different ages and sexes, we investigated how grooming time was allocated across body regions (normalized for surface area), how grooming is structured temporally, and how these patterns vary within and between individuals. Specifically, we investigate which body areas are given the most attention, when correcting for differences in surface area, and how the time allocation differs between grooming sequences and between individuals. Using hierarchical cluster analyses and a Markov chain sequence analysis, we further mathematically quantify similarities and differences between the succession of sequences. Rather than assuming and testing fixed rules, this approach allows us to describe the degree of regularity and variability in grooming organization in a data‐driven way. Finally, we consider the implications of this approach for the ethical 3R principles for performing more humane animal research (replacement, reduction, refinement): By examining the relation between within‐ and between‐individual variation, we seek to explore the potential for reducing sample size and therefore for more efficient and ethically responsible sampling strategies in future studies.

## METHOD

### Animals and husbandry

Grooming performance was recorded from a total of 17 domestic cats at the animal shelter in our city. Nine female and eight male cats, aged 9 months to 8 years, were the subjects of the study (see Supporting Information A, Table S1 for Cat ID, age, and body mass of the individuals). All cats were vaccinated, microchipped, neutered, and showed no signs of ectoparasitic infestation. They underwent weekly veterinary check‐ups. Cats were fed wet food twice a day by the shelter staff and had access to dry food and water ad libitum.

The animals were housed in rooms with one to five cats of mixed sexes and ages based on compatibility and tolerance between the cats. The rooms were cleaned daily. Indoor rooms (7.81–25.90 m^2^, ceiling height: 2.75–2.90 m) were equipped with multiple scratching posts, blankets, litter boxes, and toys (see Supporting Information A, Figure S1, Panels a and b). Most of the rooms provided access to an outdoor area (11.80–24.00 m^2^, ceiling height: 2.10 m) via a cat flap. The outdoor areas were also equipped with multiple climbing posts, hiding places, and toys (Figure S1, Panels c and d). Indoor rooms had windows on one side, which led to varying light conditions depending on the weather and orientation of the rooms.

The selection of observed cats was primarily based on the possibility of them being filmed. Because some of the cats at the shelter were too timid, retreating from grooming when observed and making filming impossible, not all of them could be included in the analysis. Therefore, the study is biased toward animals that were more acclimated to human presence.

The animals were kept in accordance with International and National animal welfare regulations. The recordings were conducted in a noninvasive manner under routine housing conditions and did not require registration as animal experiments at the local Committee for Animal Research. Approval of the recordings was granted by the animal shelter management.

### Video recordings

Videos were recorded using a standard video camera (JVC Camcorder GC‐PX100BE) at a frame rate of 50 frames per second. The filming took place once a week over a period of 10 weeks from April to June 2023 between 9:30 a.m. and 5:00 p.m. Data collection followed an event‐based observational approach. Recording was started manually when grooming activity was observed or anticipated, allowing the capture of grooming episodes as they occurred. This approach is comparable to ad libitum and focal event sampling methods (Altmann, 1974).

To ensure the best perspective for later analysis, the observer was present in the rooms with the cats during recording, which occasionally led to distractions of the animals. As recordings started upon detection of grooming, a few seconds may be missing from the beginning of some of the videos. However, these missing seconds could be reconstructed later through written notes. In addition to the video recordings in cases where grooming activity was observed but filming was not possible due to the cat's position, the duration was manually documented. This allowed for nonrecordable sequences to be included in the analysis of sequence duration.

### Procedure

#### Data collection and sample composition

Video analysis was conducted using the Kinovea 0.9.5 software (Charmant & Contributors, 2021). In total, 111 recordings were obtained (Sample A, Table 1). A subset of 40 recordings from 10 cats (Sample B, Table 1), ranging in duration from 00:42 to 17:40 min, were further evaluated for sequence structure. Video selection was based on duration and the available number of recordings per cat. For a subset of seven cats (Sample C, Table 1), we obtained at least five recordings of multicomponent grooming bouts per cat in which multiple targets on the body surface are addressed and episodes are repeated. Efforts were made to ensure that the relevant videos came from at least two different recording days. The analysis was conducted sequentially. Supporting Information B (video) shows an example of how a sequence recording was transmitted into the protocol of grooming episodes. Based on these protocols, the singular movements and postures were described and temporal and spatial parameters were extracted.

**TABLE 1:** Analysis of sequence structure and variance: Methods, sample subset, sample size (records), and sample composition.

Analysis	Sample	Cats
Description of episodes and overall grooming performance	A (*n* = 111)	All cats (ID 1 to ID 17)*
Measurement of sequence duration	A (*n* = 111)	All cats (ID 1 to ID 17)
Comparison of sequence structure by the method of event pairing and hierarchical cluster analysis; test for cephalocaudal succession of episodes	B (*n* = 40)	10 cats (IDs 1, 2, 3, 6, 8, 9, 10, 11, 13, 16)
Test for the existence of nonrandom coupling between two episodes by first‐order Markov chain sequence analysis	B (*n* = 40)	10 cats (IDs 1, 2, 3, 6, 8, 9, 10, 11, 13, 16)
Analysis of time sharing between grooming targets and body regions, analysis of variance	C (*n* = 35)	7 cats (IDs 1, 2, 3, 6, 9, 10, 13)

* see TABLE S1 for information about the individuals (age, sex, body mass).

#### Definition of grooming sequences, episodes, grooming targets, and effectors

Grooming sequences are subdivided into units called grooming episodes. An episode is defined as the interplay between a particular end effector (mouth, fore paw, hind paw) and a particular target on the body surface. Grooming targets were defined as discrete regions on the body surface. They were delineated based on careful observations of grooming performance variation, considering that grooming movements tended to be directed to specific body areas in a temporally segregated manner, indicating a natural partitioning. The partitioning further represents functional differences in the demands on body mobility and kinematics required to reach the targets. Therefore, grooming targets define functionally distinct categories (Figure 1). Nevertheless, several grooming movements still cover more than one area. In these cases, the duration of the episode was divided by the number of grooming targets included to calculate the share of each grooming target in the episode. For parts of the analysis, the grooming targets were grouped into six body regions to later test for a cephalocaudal progression and rules concerning the overall succession (Figure 1).

**FIGURE 1:**
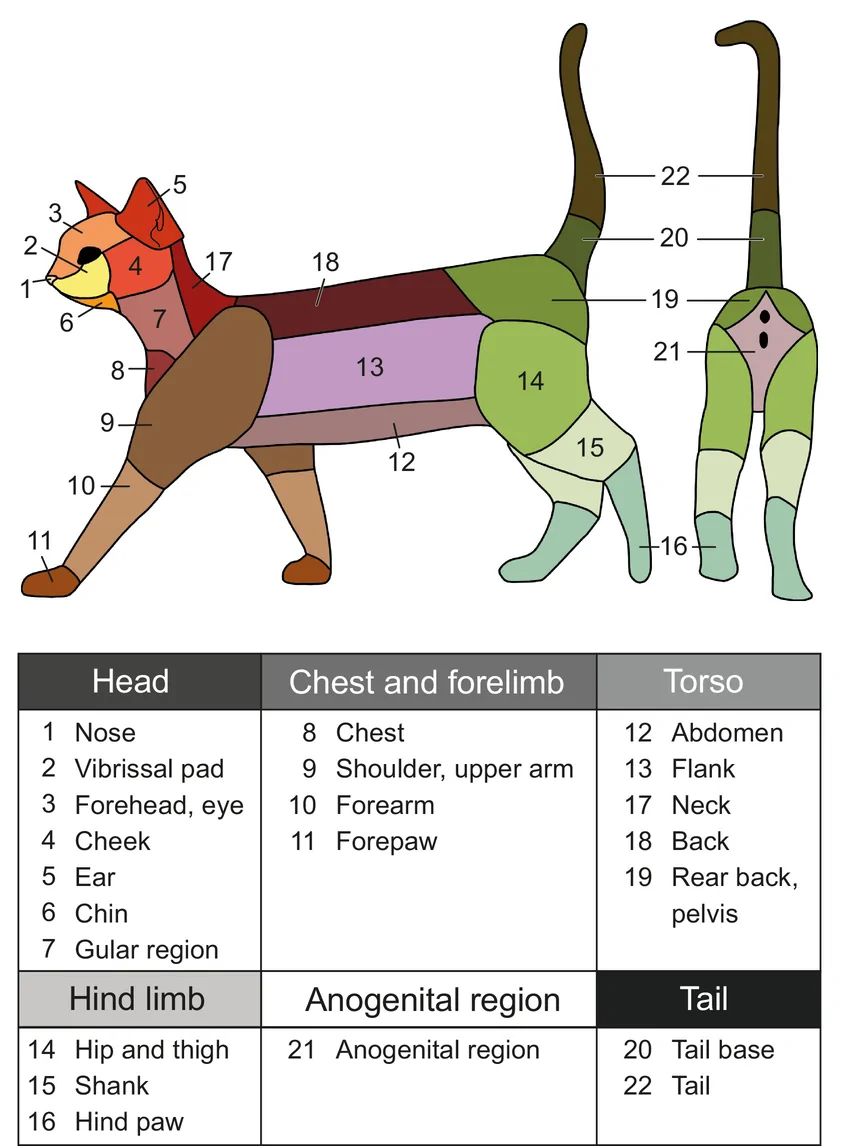
Definition of grooming target areas and body regions of the cat's body surface in lateral and posterior perspective.

The end of a sequence was defined following Eckstein & Hart (2000a): No grooming behavior was observed for at least 60 s or the animals engaged in a different activity. A change in grooming target, grooming effector, or body posture as well as interruptions of at least 0.5 s marked the end of an episode. Pauses of at least 0.5 s were categorized as such. Shorter interruptions, after which grooming of the same target with the same effector resumed, were considered part of the same episode. Short pauses sometimes occurred between two episodes, bound by the last contact of the grooming effector with the previous target and the first contact with the subsequent target. In such cases, the temporal midpoint was used as the end/beginning of the episodes if the gap between episodes was under 0.5 s. Longer interruptions were also considered as pauses. If applicable, the body side was recorded for both the grooming target and effector. In cases where grooming strokes occurred centrally, they were attributed to both sides of the body. Additionally, the tip of the nose, tail, tail base, and anogenital region were defined as sideless body regions.

### Data analysis

Sequence duration was measured for all sequences (Sample A). An ANOVA was conducted to test for differences in average sequence duration between the cats using R 4.1.3 (R Core Team, 2023).

The relative time spent on grooming different grooming targets and body regions was calculated from the sequence protocols (Sample C). Similarly, the proportions of grooming dedicated to oral grooming, face washing, and hind paw scratching were determined. These values were computed both as percentages of the total analyzed grooming time and individually for each cat. Variance in time allocation was calculated as the coefficient of variation both between cats and between the five bouts of each individual to test whether variance was greater between cats or within individual cats.

The temporal proportions of grooming targets for each sequence were normalized to their surface proportion of the cat's body to allow for better comparability of grooming targets. For this purpose, a virtual 3D‐surface model was created from a taxidermy preparation of a male domestic cat using the software Agisoft Metashape (professional version 1.4.5, 2018). Using this model, the areas of each grooming target were estimated. Their proportions of the cat's total surface area are given in Table 2.

**TABLE 2:** Share of each grooming target on total body surface relative grooming time as a percentage of total grooming time, and ratio of relative grooming time to the target's share of the body surface across the seven cats in Sample C.

Grooming target	Share of total body surface [%]	Relative grooming time [%]	Time / surface ratio
1 Nose	0.77	0.28	2.75
2 Vibrissal pads	1.40	1.07	0.76
3 Forehead, eyes	2.26	0.82	0.36
4 Cheeks	0.99	1.09	1.10
5 Ears	3.91	0.75	0.19
6 Chin	0.89	0.15	0.17
7 Gular region	2.87	0.30	0.10
8 Chest	1.23	2.15	1.74
9 Shoulders, arms	11.34	11.97	1.06
10 Forearms	6.71	11.36	1.69
11 Forepaws	2.64	9.82	3.72
12 Abdomen	5.29	8.44	1.60
13 Flanks	10.45	8.56	0.82
14 Hips and thighs	8.52	10.29	1.21
15 Shanks	4.42	4.63	1.05
16 Hind paws	7.40	4.97	0.67
17 Neck	2.99	0.07	0.02
18 Back	9.59	4.47	0.47
19 Rear back, pelvis	6.67	4.00	0.60
20 Tail base	2.37	3.70	1.56
21 Anogenital region	1.92	4.90	2.55
22 Tail	6.02	5.70	0.95

To test for a cephalocaudal succession, the six body regions were arranged in a cephalocaudal order and assigned corresponding expected ranks from 1 to 6. For each sequence, the ranks were determined for the observed grooming order. The expected and the observed ranks were tested for correlation using the Spearman method using R 4.1.3 (R Core Team, 2023).

The observed ranks were further used to quantify similarities and differences in the order of the grooming sequences by the method of event pairing (see Supporting Information A, Table S2, for a calculation example). In short, for each sequence, a matrix was created and each cell in the matrix was assigned to one of four values as shown by this example:

- 0: Rank (Body region A) < Rank (Body region B).
- 1: Rank (Body region A) = Rank (Body region B).
- 2: Rank (Body region A) > Rank (Body region B).
- NA: At least one of the body regions is not groomed in the sequence.

The cell contents of each matrix were converted into a numerical sequence, which served as the basis for comparing the grooming sequences. Those were used to compute Hamming distances between all sequences in R (R Core Team, 2023; van der Loo, 2014), which were used to conduct a hierarchical cluster analysis. Hamming distances are a measure used to quantify differences between two sequences of equal length, indicating the number of positions with different values. The greater the Hamming distance between two strings, the more different they are. The Hamming distances of the overall data set in Sample B were tested for differences from those of individual cats, sexes, and sequences with the same outcome for the cephalocaudal order using the Wilcoxon rank‐sum test in R (R Core Team, 2023).

A first‐order Markov chain sequence analysis was conducted to test for nonrandom coupling between two grooming episodes (Cane, 1978; Lehner, 1998). To exclude the influence of unequal frequency distributions of individual grooming targets across sequences, the absolute frequencies in which two events directly succeeded one another were entered into a transition matrix. Frequencies refer to the number of sequences in which the successions could be observed. Using the transition matrix and Equation 1, the expected frequency (*E*) of two grooming episodes occurring sequentially was determined, assuming that the probability of grooming a target is independent of grooming the preceding target:

(1) $$E=\frac{\text{rowsum}\left(\text{Grooming}\;\text{Target}\;1\right)*\text{columnsum}\left(\text{Grooming}\;\text{Target}\;2\right)}{\mathrm{sum}\;\text{total(grooming}\;\text{target}\;\text{frequencies)}}.$$

In this case, Grooming Target 1 indicates the preceding grooming target, Grooming Target 2 the following grooming target. The denominator gives the total sum of frequencies of all determined pairs of grooming episodes. Nonrandom couplings of two episodes can be assumed for a quotient of observed (*O*) and expected frequency (*E*) > 1 (*C*_1_; Equation 2).

(2) $$C_{1}=\frac{O}{E}.$$

To minimize the influence of erroneous results and to identify significant and highly significant couplings, the confidence level (*C*_2_) and significance level (*C*_3_) were calculated subsequently using Equation 3 and Equation 4. Again, values > 1 were considered a prerequisite for significance.

(3) $$C_{2}=\frac{O}{E+2\sqrt{E}}.$$

(4) $$C_{3}=\frac{O}{E+10\sqrt{E}}.$$

## RESULTS

The focus of the current study was to quantifiably capture the diversity in the structure and organization of feline grooming sequences. After a brief description of overall grooming performance, we analyze the variation of sequence duration, temporal proportion of effectors used for grooming, and time allocation to different grooming targets and body regions. Finally, pattern and sequence analyses are presented to identify possible rules and structure in the spatiotemporal succession of grooming episodes.

### Grooming performance in domestic cats

Most grooming time was spent on oral grooming, followed by face washing and hind paw scratching (Figure 2). Oral grooming was part of all analyzed sequences, whereas face washing was seen in 60% and scratching in about 28% of the sequences. Cats clean their face using wet forepaws and, more frequently, using the wet forearm (Figure 3a). Face washing itself consisted of mostly elliptical strokes of the forelimbs over the head, where several grooming targets were often connected by a single grooming stroke. Such strokes typically started with a grooming target at the front of the head (vibrissal pad or nose), continued posteriorly, and ended anteriorly again after a change in direction. For cleaning the ears and cheeks, smaller circular movements were observed. A maximum of three elliptical or circular strokes were performed for face washing before rewetting the forearm and paw. With few exceptions, only unilateral strokes were involved in face washing and, mostly, only one half of the head was cleaned simultaneously.

**FIGURE 2:**
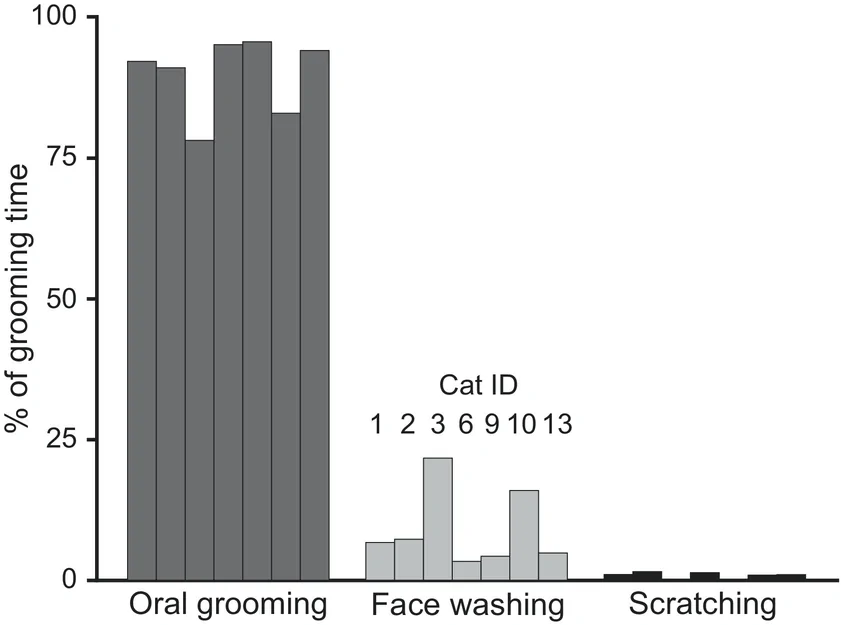
Time share of oral grooming, face washing, and scratching on total grooming time shown for seven cats (Sample C), analyzed across five sequences.

**FIGURE 3:**
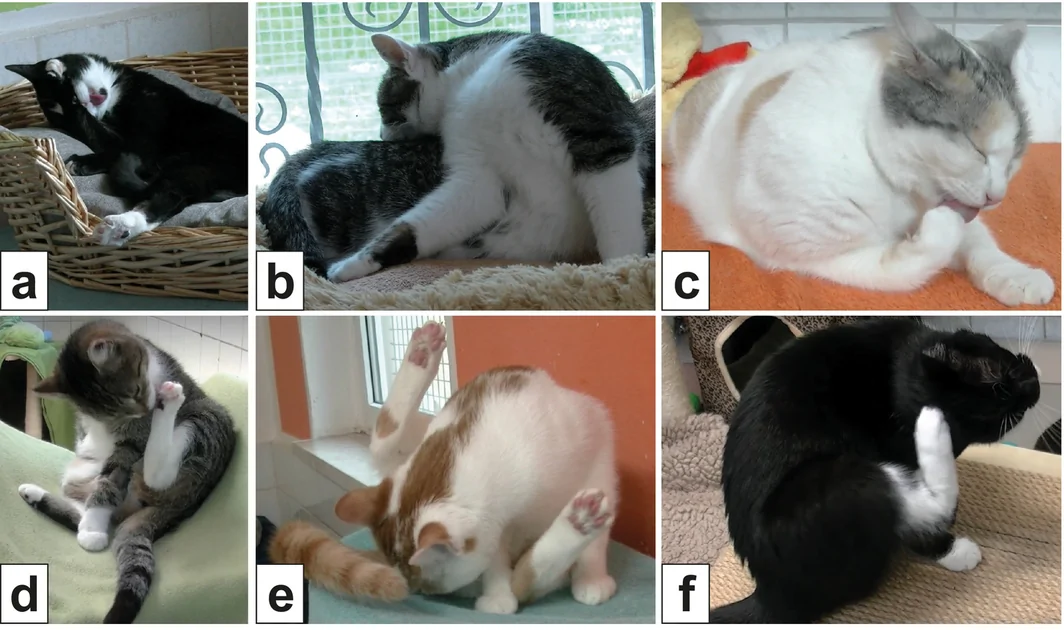
Grooming performance: Body postures adopted for face washing (a), oral grooming (b–e), and hind paw scratching (f).

Due to the high flexibility of their body axis and limbs, cats reach almost all postcranial targets with their mouth for oral grooming (Figure 3b–e), which involves both licking with the tongue and nibbling with teeth. In our observations, all orally groomed areas are cleaned from up to five but at least two of seven different postures. We found that a highly flexible spine, especially in its anterior part, was essential for all oral grooming activities (Figure 3b–e). However, high ranges of limb motions were observed less frequently: The forepaw was always lifted at least slightly and rotated to clean the palmar side (Figure 3c), whereas the hind limb was often stretched upward or forward for grooming. Due to the limited hind paw rotation in cats, the plantar side of the foot was cleaned with the hind limb strongly flexed (Figure 3d). Additionally, one or both hind limbs were always lifted to groom the anogenital region or the tail. The anogenital region appeared to be the most stereotypically groomed area, being predominantly cleaned in the same posture (Figure 3e).

Hind paw scratching is the fastest movement of all. Targets are the ears, the vibrissal pad, and the cheek as well as chin, gular region, and neck (Figure 3f). The scratching movement involved the entire hind limb performing wave‐like rotating motions. This caused the hind paws to move in rapid strokes from posterior to anterior over the grooming target.

Cats assumed a variety of body postures during grooming, with sitting and lying positions being the most common, although standing postures were also observed (Figure 3a–f). Assistive movements, which facilitate grooming without directly contributing to it, included stabilizing the position or using the forepaw to fixate the grooming target, preventing it from being passively moved during grooming strokes. We provide a more detailed description of postures and movements in Supplementary Material A (Table S2) for interested readers.

### Variation in sequence duration

The analysis of sequence duration included all analyzed sequences (Sample A). Overall, grooming sequence duration ranged from 00:21 to 17:40 min, but most of the sequences were rather short, lasting less than 3 min (Figure 4). Only eight sequences lasted longer than 10 min. There are no significant differences between the individual cats in terms of sequence duration (*p* = .242).

**FIGURE 4:**
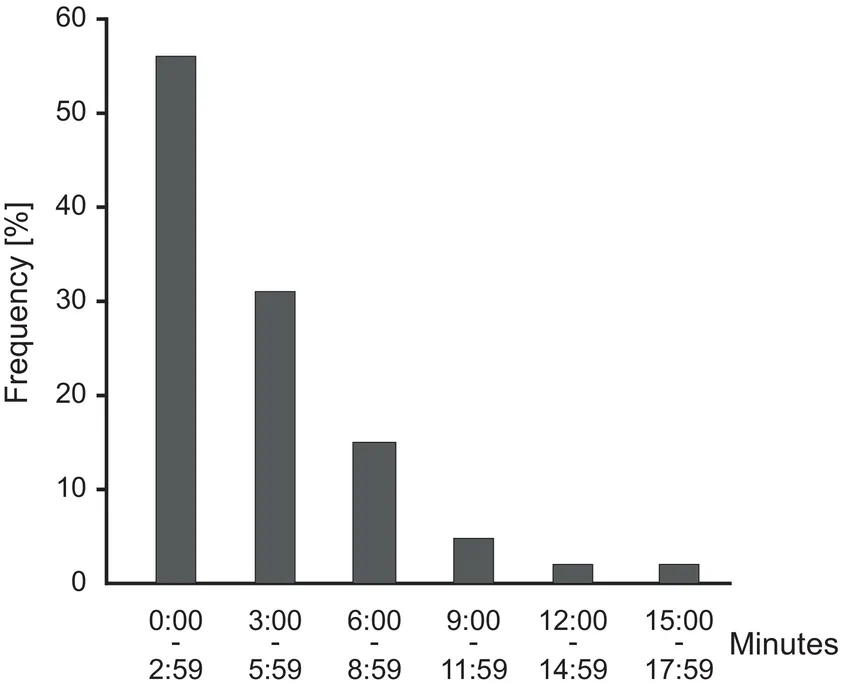
Frequency distribution of grooming sequence duration (Sample A, *n* = 111).

### Time shares of grooming targets

Due to differences in grooming sequence duration, the time shares of grooming targets were calculated as a percentage of the total analyzed grooming time of the 35 sequences in Sample C (Table 2). Consequently, a single value was obtained for each grooming target and variance analysis was conducted within individual grooming sequences. Figure 5 illustrates the distribution of grooming time to the 22 grooming targets across the seven cats (Figure 5a) and for each individual (Figure 5b–h). Common to all cats is that the chest (Target 8) and the forelimbs (Targets 9–11) receive the highest proportion of grooming time, followed by the torso (Targets 12–13), the hind limbs, and the tail. The head (Targets 1–6) and the neck (Target 7) receive remarkably less attention. In addition to this general trend, there are relatively large differences between the cats and between the individual sequences in each animal. Some cats like Cat ID 1 (male), 2, and 10 (both females), groomed nearly all targets in most of the recorded bouts, whereas other individuals like Cat ID 9 and Cat ID 13 (both males) were apparently less engaged in covering every target in every bout. However, for all but the forepaws (Target 11), the within‐cat variation exceeds the between‐cat variation (Figure 6).

**FIGURE 5:**
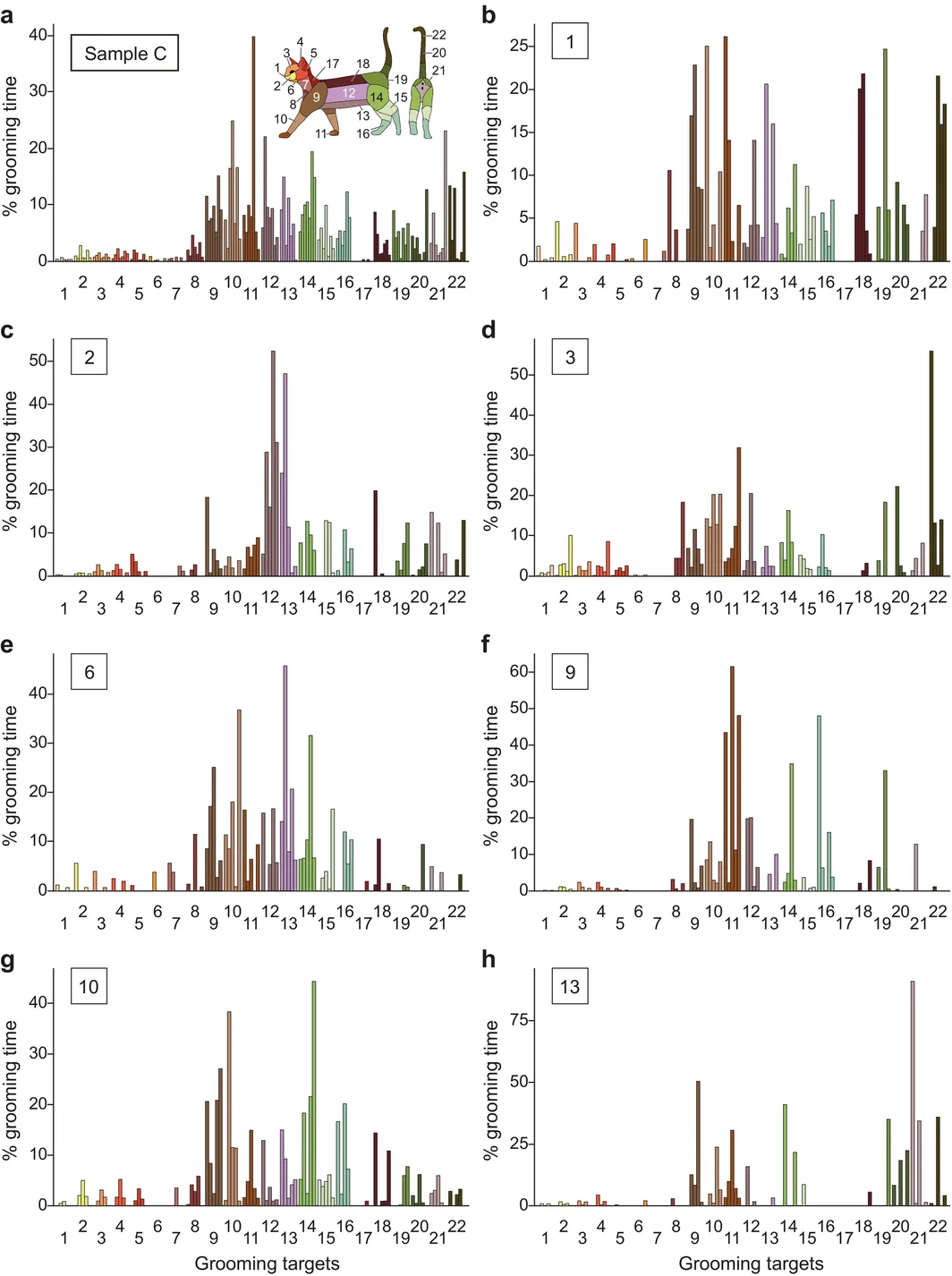
Distribution of grooming time to grooming targets: (a) shows the overall temporal distribution across seven cats (Sample C), each analyzed over five sequences, and (b–h) show the temporal distribution for each sequence of those seven cats individually.

**FIGURE 6:**
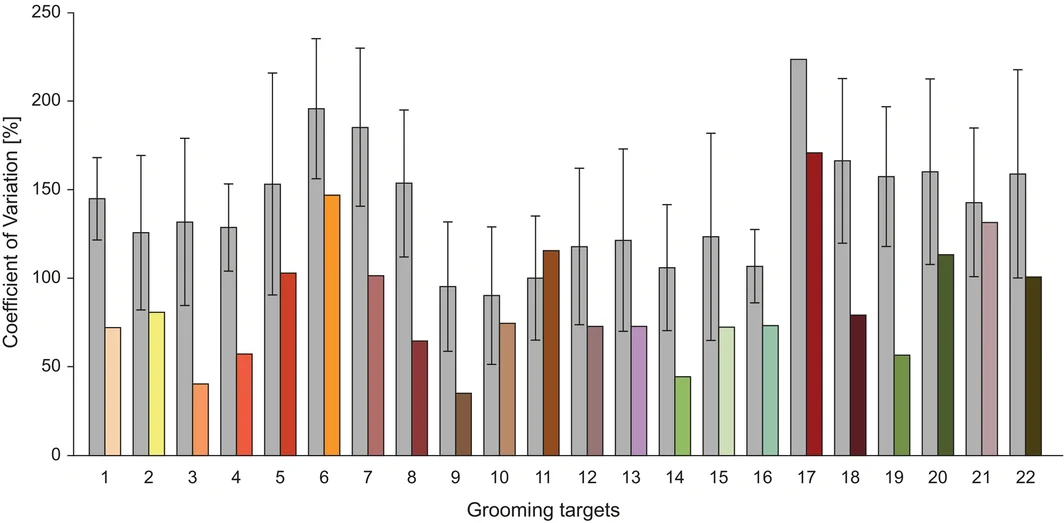
Variation of grooming time for each grooming target, expressed as the coefficient of variation (CV). Light gray bars represent within‐cat variation (calculated from five sequences for each of seven cats, Sample C), and colored bars represent the between‐cat variation (across the seven cats). Except for Grooming Target 11 (forepaws), the mean within‐cat variation is higher than the between‐cat variation.

When grooming time was normalized to the surface area of the targets (Table 2), the relative grooming time dedicated to the targets differ from the absolute values in such a way that the face (Targets 1–4), the forepaw, and the anogenital region receive more attention. The relative time spent on grooming the proximal parts of the limbs is lower due to their higher proportion of the body surface area.

### Spatiotemporal structure of grooming sequences

To test for a directional trend in the sequence of grooming episodes across the six body regions, a Spearman correlation test was conducted. The head was considered the sole target of face washing, so face washing was treated as a unit with an expected rank of 1. Because hind paw scratching, mostly targeted at the head, could not be observed at a specific point during a sequence, it was excluded from this analysis. Therefore, the spatiotemporal structure refers to only oral grooming and face washing. The Spearman correlation coefficient differed from zero in all but four sequences (Figure 7). A positive correlation, indicating a cephalocaudal progression, was obtained for 22 of the 40 sequences, three of which were significant (*p* < .05). A negative correlation, indicating a progression of grooming episodes from caudal to cranial, was revealed for 14 sequences, one of which was significant.

**FIGURE 7:**
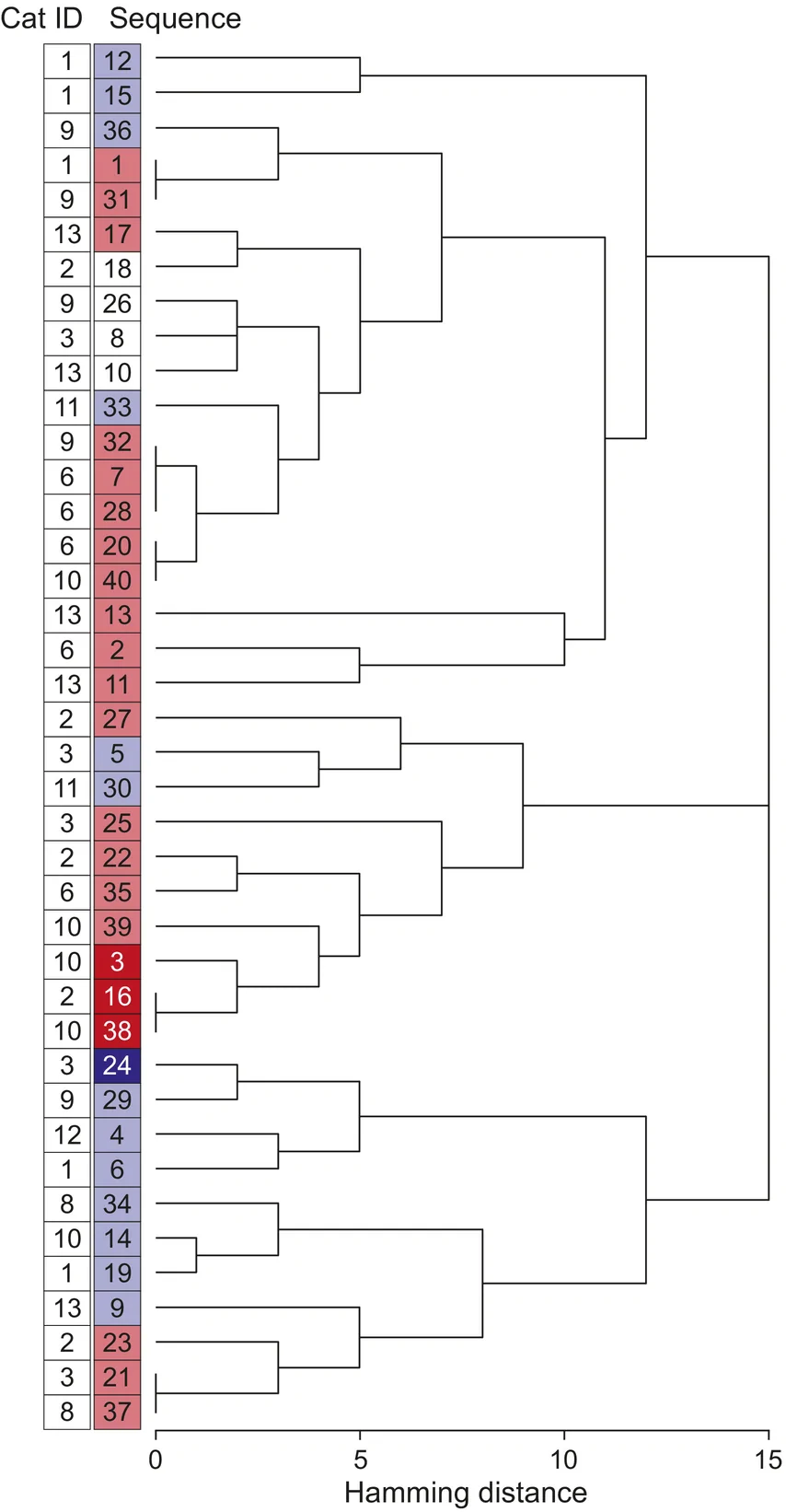
Dendrogram illustrates similarities between sequences in terms of the order of body regions groomed in the sequences; small Hamming distances indicate a similar spatial and temporal succession of sequences. Sequence box marked in dark red: Spearman correlation significantly positive (cephalocaudal progression), light red: not significantly positive, white: neutral, dark blue: significantly negative (caudocephalic progression), light blue: not significantly negative.

Next, we used the event‐pairing matrix to quantify Hamming distances as a measure of similarity between two sequences in the entails of their spatiotemporal structure. Figure 7 shows the resulting dendrogram. It supports our finding of a less strict cephalocaudal succession of grooming episodes in the sequences of our sample. Although sequences with the same directional trend are more similar to each other than to the average (*p* < .005), the details of the spatiotemporal coupling of grooming regions have a greater overall influence on the degree of similarity. It means that even two sequences with different directional trends can share a low value for Hamming distance and thus can be more similar to each other in the details of their structure. Furthermore, similar sequences do not necessarily belong to the same cat. The sequences of three cats (ID 2, 3, 13) are distributed across all three main branches in the dendrogram, the sequences of the other four cats are distributed across two main branches. No differences were asserted between the overall mean Hamming distance of 9.5 and the Hamming distances of the individual cats (*p* values range from .077 in ID 3 to .878 in ID 2). Equally, the Hamming distances of female and male cats do not differ from the mean of the overall data set (females: *p* = .883; males: *p* = .209).

The Markov chain model was the next step in our analysis of the spatiotemporal structure of grooming sequences, now with the focus on the sequence of episodes directed to the targets. Markov chain sequence analyses are established tools in behavioral research that enable the identification of rules in sequences of events. We used them to identify significant and highly significant couplings between pairs of grooming targets (Figure 8). The transition matrix contains values for a total of 240 possible pairs of grooming targets and considers the 14 orally groomed targets. Face washing was considered here as a single unit because our observations could not fully resolve the temporal order of grooming episodes across the face in many sequences. Also, hind paw scratching regardless of the target is considered as a single unit. Thus, the total number of considered Markov chains is 792. Of the 240 possible transitions, 66 were not observed in any of the sequences. Nonrandom coupling was determined for 99 pairs of grooming targets (*C*_1_ > 1). Significant coupling was assessed for 28 and highly significant coupling for two event pairs (anogenital region ⇒ tail base; tail base ⇒ tail). As illustrated in Figure 8, many couplings are bidirectional, which again highlights that there is no strict cephalocaudal succession of grooming episodes. Across the postcranial body surface, significant coupling occurs between neighboring targets. Also significant is the unidirectional coupling between face washing and oral grooming of the forepaw or the forearm, a movement sequence that everyone would consider as most typical for grooming in cats.

**FIGURE 8:**
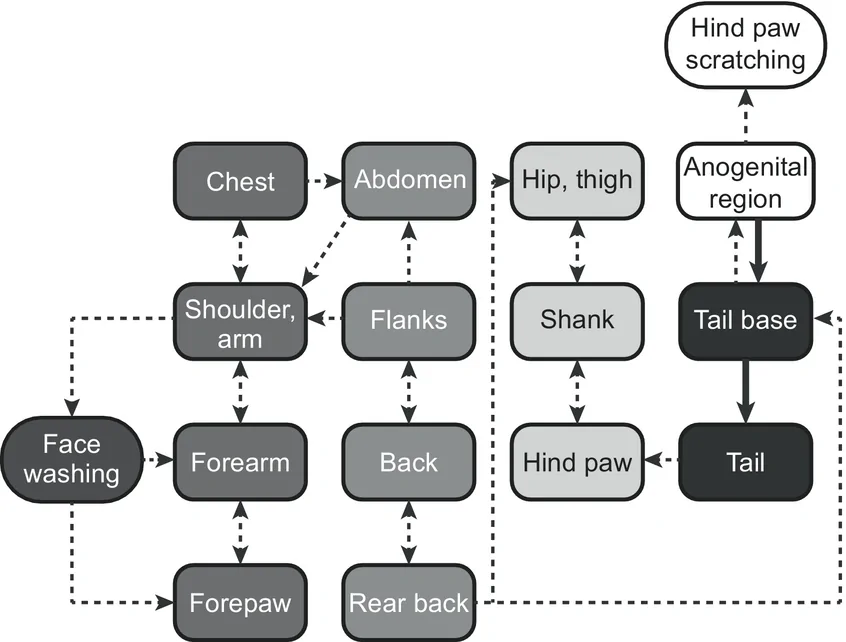
Markov chain sequence analysis: The flow diagram illustrates significant (dashed arrows) and highly significant (bold arrows) couplings between the grooming of two grooming targets.

## DISCUSSION

Our results reveal a high diversity in the structure and organization of self‐grooming in domestic cats. They provide insights into the temporal distribution of grooming activity across different body areas as well as the individual animals' preferred way of grooming (oral grooming, face washing, hind paw scratching). High variation is evident across all observed levels of the cats' grooming process. This encompasses not only the extensive repertoire of postures and movements involved in grooming but also variability in sequence duration, differing temporal distribution across body areas, and diversity in terms of the spatial and temporal succession of grooming sequences.

Domestic cats primarily use oral grooming for body care. Self‐grooming of a specific grooming target was carried out either by alternating between relatively long episodes of body licking and short episodes of “nibbling” or exclusively by the tongue. The overall high contribution of body licking can be interpreted in the context of its crucial role in distributing saliva across the body surface, which aids in thermoregulation (Noel & Hu, 2018). Cats dedicate about 2.4 hr per day to grooming, enabling them to dissipate 25% of their excess resting heat energy through saliva distribution, thereby protecting them from overheating (Noel & Hu, 2018). Oral body care gains significance in the context of thermoregulation because cats have sweat glands only on their paw pads (Adams et al., 1970; Noel & Hu, 2018). Other methods to dissipate excess heat, such as panting, are not sufficient to maintain a constant body temperature at high ambient temperatures (Adams et al., 1970). Smit et al. (2026) recently reported significantly higher grooming activity of domestic cats in the warmer summer months than in winter. When correcting for surface area, only minor differences can be seen in the time allocated to different body areas. Cleaning of body regions corresponding to their surface area seems reasonable for the role of thermoregulation as well as for preventing infestation with ectoparasites.

The importance of grooming in the context of parasite defense and prevention is supported by the absence of external triggers for grooming during the recordings. It is assumed that grooming behavior is an innate and stereotyped behavior controlled endogenously, which can be observed without the need for sensory feedback (Berridge & Fentress, 1987; Fentress, 1973). This is related to the regular integration of grooming sessions into the daily routine, serving as a preventative measure against parasite infestation (Berridge & Fentress, 1987). The high susceptibility to infestation with ectoparasites such as lice and ticks and with the numerous diseases these parasites can transmit (Beck et al., 2006; Beugnet et al., 2014; Bond et al., 2007; Lappin, 2018; Sotiraki et al., 2001; Thomas et al., 2016) supports the important function of grooming in feline parasite defense.

The naturally reinforcing properties of grooming may explain why cats engage in prolonged grooming bouts during which multiple body regions are covered. However, our findings suggest that this outcome does not require conserved or stereotyped grooming sequences. Instead, a high degree of variability can be observed in the spatiotemporal succession of sequences. This also applies to the commonly described cephalocaudal progression: only 22 of the 40 sequences showed a positive correlation between the grooming order of body regions and the expected rank. The correlation was significant in only three of these 22 sequences. Consequently, a craniocaudal grooming sequence cannot be assumed as a general organizational principle in cats. Instead, it suggests a random succession of grooming sequences. This stands in contrast with findings of earlier studies on different species, where such progression has been described as a characteristic feature of grooming sequences (Berridge, 1990; Fentress & Stilwell, 1973; Lefebvre, 1982; Richmond & Sachs, 1980). The sequence comparison using event pairing also does not reveal any patterns in the sequence organization. The order of body regions differs from sequence to sequence, and even sequences of the same cat or same sex are not more similar to each other than expected by chance, ruling out a systematic approach to the differences. In contrast to rodents, where grooming sequences have been described as syntactic chains with a hierarchical organization (Berridge, 1990; Berridge et al., 1987), the present results do not support the existence of fixed individual or species‐level sequence templates in cats.

The results of the Markov chain sequence analysis, however, suggest that the sequence of grooming targets is not entirely random. Several significant transition probabilities between grooming targets reveal structured patterns at a smaller local level. Notably, these couplings primarily occur within body regions or between grooming targets in direct spatial proximity. Moreover, these patterns reflect probabilistic tendencies rather than deterministic rules: Although certain transitions occur more frequently than expected by chance, they are neither obligatory nor predictive of the overall sequence structure. This contrasts with interpretations from classical neuroethological studies in which such regularities have often been taken as evidence for strict rules or fixed‐sequence organization (e.g., Berridge et al., 1987; Fentress & Stilwell, 1973). Therefore, our results of statistically significant couplings between grooming targets should not be interpreted as evidence for fixed deterministic rules. Instead, they suggest that grooming sequences in cats are regulated in a flexible “rule‐free” manner at the global level despite showing nonrandom transitions at the local level.

Similar observations of global variability combined with local structure were made by Dawkins and Dawkins (1976) for the grooming movements of blowflies (*Calliphora vincina*). The authors put forward their hypothesis under the term “postural facilitation.” This model predicts that grooming sequences are structured in a way that requires few and small changes in body posture so that grooming acts that can be done from a similar position are more likely to follow one another (Dawkins & Dawkins, 1976). The concept of postural facilitation therefore describes an organization of grooming based on energy and time efficiency. This offers a possible explanation not only for why certain grooming sequences are more likely to be observed than others but also for the integration of hind paw scratching and face washing into sequences. Cats always groomed the anogenital region with at least one rear leg raised. From this posture, it only takes a few changes to scratch grooming targets, especially in the head area. This is reflected in the increased probability of scratching to follow grooming of the anogenital region. That face washing is more likely to be observed after oral grooming of the upper forelimbs can be explained by face washing beginning with the cats wetting the adjacent grooming targets—the lower forelimb and forepaw.

Our findings suggest that grooming sequences in cats are not governed by fixed deterministic rules but instead are flexibly organized based on posture‐dependent transition probabilities. In this sense, grooming organization reflects structured yet rule‐free regulation in which patterns arise from efficiency and spatial proximity rather than from strict rules. Functionally, the naturally reinforcing properties of grooming may explain the persistence of grooming bouts, within which the coverage of multiple body areas is the consequence of continued grooming combined with a flexible but locally constrained sequence organization. Because cats can reach most grooming targets from multiple postures, numerous equally efficient grooming trajectories are possible.

### AUTHOR CONTRIBUTIONS

AH: Methodology, investigation, formal analysis, validation, writing the original draft, visualization, data curation, revision of the manuscript.

MS: Conceptualization, project administration, validation, visualization, writing the original draft, revision of the manuscript.

## CONFLICT OF INTEREST STATEMENT

No competing interests are reported by the authors.

## ETHICS APPROVAL

All procedures were performed in compliance with national and international laws and institutional guidelines.

## Supporting information


**Supporting information A** **TABLE S1:** Sample composition: Approximate age, sex, and body mass of the 17 cats observed. Total number of records and number of analysed sequences.
**FIGURE S1:** Study site. Indoor (a,b) and outdoor (c,d) rooms at the animal shelter.
**TABLE S2:** Comparison of two sequences by the method of event pairing and the computation of Hamming distances.

**Supporting information B** **VIDEO 1:** Transfer of observations into the protocol for analyzing spatio‐temporal parameters and patterns. In the plot, the upper two bars represent the left body side, and the lower two bars represent the right side. The inner two bars use color coding for grooming organs/modes (dark red: tongue; light red: teeth; orange: face washing; blue: hind paws/scratching). The outer two bars indicate the grooming target using the same colors as in the illustration on the right. The red line in the plot marks the position in the video above. In the illustration, the grooming targets groomed in the video are highlighted in red. The example depicted is a section of a sequence from a 9‐month‐old male.

## Data Availability

The raw data on duration and time distribution across grooming modes and body areas for the 40 analyzed sequences are deposited at Mendeley (DOI: 10.17632/x5c38ztg9m.1).
