# Supplementary material for: Decoding diversity: Are there patterns in the organization of grooming movements in domestic cats?
Source: J Exp Anal Behav. 2026 Oct 1;126(3):e70146. doi: 10.1002/jeab.70146 (PMC13631463; doi:10.1002/jeab.70146)
Supplement: Supplementary file 1 [file JEAB-126-0-s001.docx]

**Supporting information, A:**

**TABLE S1:** Sample composition: Approximate age, sex, and body mass of the 17 cats observed. Total number of records and number of analysed sequences.

| ***Cat ID*** | ***Age [years]*** | ***Sex*** | ***Body mass [kg]*** | ***Records*** | ***Analysed sequences*** |
| --- | --- | --- | --- | --- | --- |
| **1** | 0.75 | m | 3.4 | 10 | 5 |
| **2** | 8 | f | 5.2 | 9 | 5 |
| **3** | 3 | f | 4.9 | 6 | 5 |
| **4*** | 0.75 | f | NA | 2 | 0 |
| **5** | 0.75 | f | NA | 1 | 0 |
| **6** | 5.5 | f | 4.7 | 12 | 5 |
| **7** | 1 | f | 3.4 | 4 | 0 |
| **8** | 5 | m | 5.8 | 6 | 2 |
| **9**** | 2 | m | 5.0 | 11 | 5 |
| **10** | 5 | f | 3.3 | 17 | 5 |
| **11** | 2 | m | 5.6 | 10 | 2 |
| **12** | 1 | m | 4.6 | 1 | 1 |
| **13** | 1 | m | 4.5 | 5 | 5 |
| **14** | 1 | m | 4.5 | 1 | 0 |
| **15** | 1 | m | 4.7 | 4 | 0 |
| **16** | 1 | f | 3.6 | 8 | 0 |
| **17** | 5 | f | 4.3 | 4 | 0 |

*Selkirk Rex mixed breed with curled hairs. **The cat showed sometimes unusual head movements.

**FIGURE S1:** Study site. Indoor (a,b) and outdoor (c,d) rooms at the animal shelter.

**a**

**b**

**c**

**d**

**TABLE S2:** Comparison of two sequences by the method of event pairing and the computation of Hamming distances.

Step 1: Calculate ranks (=mean position in sequence) of body regions in each sequence:

| Sequence 27 | |  | Sequence 29 | |
| --- | --- | --- | --- | --- |
| **Body region** | **Rank** |  | **Body region** | **Rank** |
| Head (A) | 10.00 |  | Head (A) | 13.00 |
| Chest and forelimb (B) | 14.50 |  | Chest and forelimb (B) | 11.83 |
| Torso (C) | 9.57 |  | Torso (C) | 10.50 |
| Hind limb (D) | 14.67 |  | Hind limb (D) | 7.40 |
| Anogenital region (E) | 14.00 |  | Anogenital region (E) | 6.50 |
| Tail (F) | 11.33 |  | Tail (F) | 8.00 |

Step 2: Set up matrix to compare relative occurrence of body regions *within* each sequence:

Sequence 27:	Sequence 29:

|  | A | B | C | D | E | F |  |  | A | B | C | D | E | F |
| --- | --- | --- | --- | --- | --- | --- | --- | --- | --- | --- | --- | --- | --- | --- |
| B | 0 |  |  |  |  |  |  | B | 2 |  |  |  |  |  |
| C | 2 | 2 |  |  |  |  |  | C | 2 | 2 |  |  |  |  |
| D | 0 | 0 | 0 |  |  |  |  | D | 2 | 2 | 2 |  |  |  |
| E | 0 | 2 | 0 | 2 |  |  |  | E | 2 | 2 | 2 | 2 |  |  |
| F | 0 | 2 | 0 | 2 | 2 |  |  | F | 2 | 2 | 2 | 0 | 0 |  |

Step 3: Transform matrices into strings of numbers to compare order of body regions *between* sequences:

|  | A-B | A-C | B-C | A-D | B-D | C-D | A-E | B-E | C-E | D-E | A-F | B-F | C-F | … |
| --- | --- | --- | --- | --- | --- | --- | --- | --- | --- | --- | --- | --- | --- | --- |
| Sequence 27: | 0 | 2 | 2 | 0 | 0 | 0 | 0 | 2 | 0 | 2 | 0 | 2 | 0 |  |
| Sequence 29: | 2 | 2 | 2 | 2 | 2 | 2 | 2 | 2 | 2 | 2 | 2 | 2 | 2 |  |

Step 4: Calculate Hamming distances as the number of different positions in the string for each possible pair of sequence.

In this example the Hamming distance between sequence 27 and 29 is 10, because 10 out of the 15 positions differ between the two strings.
